# Development of Precision Controllable Magnetic Field-Assisted Platform for Micro Electrical Machining

**DOI:** 10.3390/mi15081002

**Published:** 2024-08-01

**Authors:** Cheng Guo, Weizhen Zhuang, Jingwen He

**Affiliations:** 1 Guangdong Provincial Key Laboratory of Micro/Nano Optomechatronics Engineering, College of Mechatronics and Control Engineering, Shenzhen University, Shenzhen 518060, China; 2310295112@email.szu.edu.cn (W.Z.); 2100291001@email.szu.edu.cn (J.H.); 2 Shenzhen Key Laboratory of High Performance Nontraditional Manufacturing, College of Mechatronics and Control Engineering, Shenzhen University, Shenzhen 518060, China

**Keywords:** magnetic field, micro electrical machining, electromagnetic coil, spherical harmonic function, magnetic multipole superposition

## Abstract

In order to introduce the magnetic field into micro electrical machining technology to explore the influence of magnetic field on micro electrical machining, the development of a precision controllable magnetic field-assisted platform is particularly important. This platform needs to precisely control the spatial magnetic field. This study first completes the hardware design and construction of the magnetic field generation device, using electromagnetic coils with soft iron cores as the sources of the magnetic field. Mathematical models of the magnetic field are established and calibrated. Since the magnetic dipole model cannot effectively describe the magnetic field generated by the electromagnetic coil, this study adopts a more precise description method: the spherical harmonic function expansion model and the magnetic multipole superposition model. The calibration of the magnetic field model is based on actual excitation magnetic field data, so a magnetic field sampling device is designed to obtain the excitation magnetic field of the workspace. The model is calibrated based on a combination of the theoretical model and magnetic field data, and the performance of the constructed setup is analyzed. Finally, a magnetic field-assisted platform has been developed which can generate magnetic fields in any direction within the workspace with intensities ranging from 0 to 0.2 T. Its magnetic field model arrives at an error percentage of 2.986%, a variance of 0.9977, and a root mean square error (RMSE) of 0.71 mT, achieving precise control of the magnetic field in the workspace.

## 1. Introduction

Micro electrical machining has the advantages of non-contact processing and high precision, which can significantly reduce the possibility of workpiece deformation and surface damage, and is an ideal method for the fabrication of micro-structures and precision components. However, there are also some shortcomings in micro electrical machining, such as low processing efficiency (micro-EDM), poor localization, and serious stray corrosion (micro-ECM). Magnetic field assistance may improve the machining performance. In order to introduce the magnetic field into micro electrical machining technology to explore the influence of magnetic field on micro electrical machining, it is particularly important to develop a precision controllable magnetic field-assisted machining platform.

For magnetic field generation devices, there are generally two types of magnetic field sources: permanent magnets and electromagnetic coils [1]. The setup based on permanent magnets generate the target magnetic field intensity by changing the posture of the permanent magnet. Currently, most of them are implemented by installing a permanent magnet at the end of robotic arms, which can generate magnetic fields with larger magnetic field gradients. The magnetic field platforms based on electromagnetic coils are mostly constructed with electromagnetic coils and soft magnetic cores. By changing the driving current of the electromagnetic coil and the layout of multiple electromagnetic coils, the target magnetic field is generated.

Shanghai Anhan Medical Technology Limited Liability Company [2] has developed a capsule endoscope robot drive device based on a permanent magnet. The permanent magnet is installed on a moving platform with two rotational degrees of freedom and three translational degrees of freedom. The magnetic field intensity in the workspace is controlled by the movement and rotation of the platform. Ciuti et al. [3] installed a cylindrical permanent magnet at the end of a six-degree-of-freedom industrial robotic arm (RV-3SB), and achieved effective interaction between the permanent magnet and the controlled device. This device can achieve up to two translational and two rotational degrees of freedom. The magnetic field sources of the above-mentioned magnetic field-generating devices are all single permanent magnets, which makes it difficult to obtain high-uniformity magnetic fields. Some researchers have begun to use multiple permanent magnets as magnetic field sources [4,5,6]. Among them, the most representative is the Niobe system [7] developed by Stereotaxis Inc. in the US in 2003. This system distributes two permanent magnets on both sides of the workspace housing and controls the magnetic field parameters of the workspace by rotating the housing, achieving precise magnetic field control. Specific magnetic fields can be realized by using multiple reasonably distributed permanent magnets. Zarrouk et al. [8] placed four bar magnets in a fixture and mounted the assembly on a commercial robotic arm. This setup can generate strong magnetic fields because the magnetic field source is a permanent magnet. However, the overall size and weight of the device are relatively large, which is costly in production and maintenance. In addition, since the magnetic field of the permanent magnet cannot be adjusted by external control, it is possible to cause accidents. Therefore, researchers are more inclined to choose electromagnetic coils as the magnetic sources.

A pair of coils can be placed facing each other. The workspace is located at the center, and the most common ones are Helmholtz coils and Maxwell coils. The setup made by different combinations of these two kinds of coils can generate arbitrary high-uniformity magnetic fields in a plane or space [9]. By applying appropriate excitation currents to each electromagnetic coil or changing the posture of the electromagnetic coil, the device can generate various magnetic fields, such as rotating magnetic fields, conical magnetic fields, and alternating magnetic fields [10,11,12]. The team of Prof. Song Shuang [13] from Harbin Institute of Technology has established a setup that combines Helmholtz coils and Maxwell coils. This device can generate controllable uniform magnetic fields in a three-dimensional space and can also generate spatial magnetic fields with different characteristics.

To improve energy efficiency and reduce layout constraints, distributed electromagnetic coils have been proposed, which typically use smaller cylindrical or rectangular coils. In addition, to enhance the magnetic field in the workspace, soft iron cores are generally added to the electromagnetic coils. After adding the soft iron core, the electromagnetic coil is more easily magnetized by the external magnetic field and can be quickly demagnetized after the external magnetic field disappears. The OctoMag [14,15] developed by Prof. Nelson’s team is the first magnetic field driving system using distributed electromagnetic coils. The setup consists of eight distributed cylindrical electromagnetic coils with soft iron cores and can realize five degrees of freedom movement of the magnetic robot. Subsequently, the team redesigned different models of MiniMag [16] based on OctoMag, which are smaller in size and offer excellent portability.

To avoid singularities and minimize the total driving current of the system, Sikorski et al. [17] designed the BigMag system. This system includes six electromagnetic coils, and the position of the six electromagnetic coils can be changed by moving the frame, thereby improving the control performance of the device. However, this also increases the complexity of establishing the magnetic field mathematical model and the driving algorithm, and the overall safety of the device decreases.

Magnetic field generation devices based on permanent magnets can generate strong magnetic fields. To generate a specific magnetic field at a particular point in the workspace, it is necessary to adjust the posture of the permanent magnets. Therefore, the overall device has moving parts, which is hard to integrate with the micro-machining platform. In addition, the magnetic field strength and direction generated by the permanent magnet are determined. By adjusting the posture of the permanent magnet, it is hard to obtain both the target magnetic field strength and direction at the same time. The flexibility of the device is relatively low. Magnetic field generation devices based on electromagnetic coils can obtain different magnetic fields by changing the driving current. The device can obtain magnetic fields of different directions and strengths without moving parts. Using multiple electromagnetic coils in combination, it is easier to generate dynamic magnetic fields and has higher flexibility.

At present, there are two methods for characterizing the magnetic field in the workspace: simulation and mathematical modeling. Simulation is simpler than mathematical modeling, and most researchers use COMSOL 5.6 simulation software for magnetic field simulation and calibration. In theory, numerical methods such as finite element methods can simulate the magnetic field distribution. However, the parameters of electromagnetic coils are difficult to control, and only specific cases can be accurately simulated. It is difficult to use numerical models to solve the target magnetic field. A more accurate magnetic field distribution can be obtained by using a mathematical modeling method. The most commonly used magnetic field mathematical model is the magnetic dipole theoretical model [9], which is based on the Biot–Savart law and the superposition principle. This modeling method is more suitable for the setup based on a permanent magnet. If the electromagnetic coil is far away from the working area, the electromagnetic coil can be simplified using an analytical magnetic dipole model. However, due to the rapid decay of the magnetic field with distance, it is difficult to economically achieve a strong magnetic field at a far distance. If the electromagnetic coil is close to the working area, the simplification conditions are not met, and the magnetic dipole model cannot be used to calculate the field distribution.

In order to achieve precise control of the magnetic field in the workspace for micro electrical machining, this study chooses cylindrical electromagnetic coils with soft iron cores as the source of the magnetic field. Since the magnetic dipole model cannot effectively describe the magnetic field generated by the electromagnetic coil, this study will adopt more accurate description methods: the spherical harmonic function expansion model and the magnetic multipole superposition model. In addition, this study will also design a magnetic field sampling device to measure the magnetic field of the workspace, so that the magnetic field model can be calibrated according to the actual excitation magnetic field data to improve the accuracy of the model.

## 2. Magnetic Field Generation Device and Sampling Device

### 2.1. Requirement Analysis of the Magnetic Field Generation Device

In magnetic field-assisted electrical machining technology, the key is the development of the magnetic field generation device. The performance of the device is directly related to the stability, analyzability, and repeatability of the experiment. The following will introduce and analyze the performance of the magnetic field generation device from different aspects.

#### 2.1.1. Strength and Flexibility

It is necessary to have magnetic fields of different intensities that can be continuously varied to more comprehensively explore the effects on micro electrical machining. The magnetic field generation device should be able to adjust the parameters of the magnetic field in real-time according to the needs, to explore the impact of the magnetic field on the micro electrical machining process. Additionally, to facilitate magnetic field-assisted electrical machining experiments, the device should be easily integrated with the electrical machining platform.

#### 2.1.2. Accuracy

In magnetic field-assisted machining, the machining location cannot provide feedback signals, so it is necessary to precisely characterize and control the direction and amplitude of the auxiliary magnetic field at the machining location. For this purpose, a reasonable mathematical model should be selected and calibrated to obtain a high-accuracy magnetic field model, and based on this model, the precise control of the magnetic field can be achieved.

#### 2.1.3. Safety

The magnetic field of a permanent magnet is generated by the material’s inherent magnetism, without electrical hazards, and the working process does not involve current conduction. The thermal effects produced during the magnetic field generation process are negligible. However, the magnetism of the permanent magnet does not disappear, which is not conducive to the installation of the workpiece during the experimental process and may pose a threat to the operators. In contrast, the magnetic field generation device based on electromagnetic coils will not produce a magnetic field without the excitation current, which can effectively ensure the safety of operators. However, the working process involves electrical equipment, and issues of overload and heat dissipation need to be considered.

Taking into account the aforementioned problems, this study will construct a magnetic field generation device based on electromagnetic coils. The goal is to generate a magnetic field with a maximum intensity of 0.2 T within the workspace, while achieving independent control of the magnetic field. Simultaneously, a more accurate mathematical model of the magnetic field will be established to minimize the impact of errors in magnetic field characterization on the results.

### 2.2. Hardware Design of the Magnetic Field Generation Device

#### 2.2.1. Electromagnetic Coil Group

This study utilizes multiple electromagnetic coils as the source of the magnetic field to optimize the distributed electromagnetic coil system. To increase the magnetic field strength in the workspace and improve energy utilization efficiency, a special arrangement is used in this study; that is, the positions of the electromagnetic coils are arranged around and directed towards the workspace center. Additionally, to enhance the magnetic strength and gradient produced by a single coil, a soft iron core is filled inside the electromagnetic coil. These types of electromagnetic coils are characterized by being easily magnetized by an external magnetic field and rapidly demagnetized.

**Figure 1:**
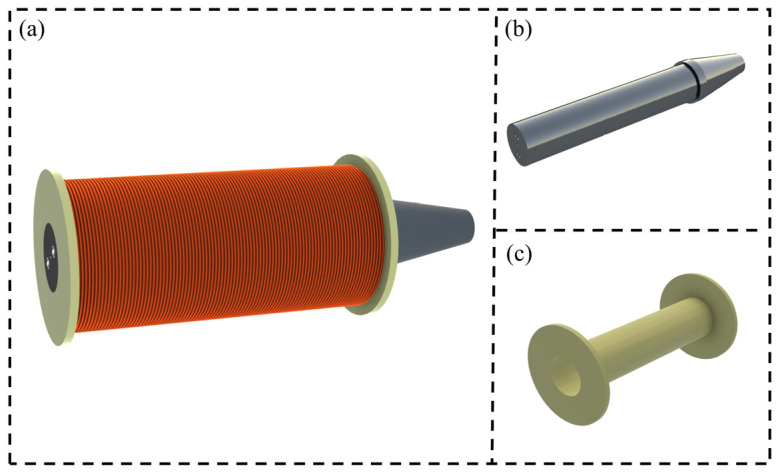
Design of a single electromagnetic coil: (**a**) model of a single electromagnetic coil; (**b**) industrial pure iron soft iron core; (**c**) keel.

Currently, the electromagnetic coils used in most magnetic field generation devices are mainly divided into cylindrical coils and rectangular coils. Compared to cylindrical electromagnetic coils, rectangular electromagnetic coils have a simpler magnetic field model establishment and require lower precision in material processing. However, the magnetic field strength produced by rectangular electromagnetic coils is lower, so they are usually used to drive micro-robots. In contrast, magnetic field systems based on cylindrical electromagnetic coils can produce stronger magnetic fields and are more widely applicable. Furthermore, industrial pure iron is selected as the material for the iron core due to its excellent magnetic permeability and strong response to the magnetic field. Additionally, industrial pure iron has a low hysteresis loop, meaning it is easily magnetized and demagnetized with high efficiency when subjected to changing magnetic fields, effectively reducing energy loss and meeting the requirements for generating dynamic magnetic fields. As shown in Figure 1, the coils are wound in a cylindrical manner around the keel, with each electromagnetic coil wound 1400 turns, and an industrial pure iron core processed and inserted into the keel.

**Figure 2:**
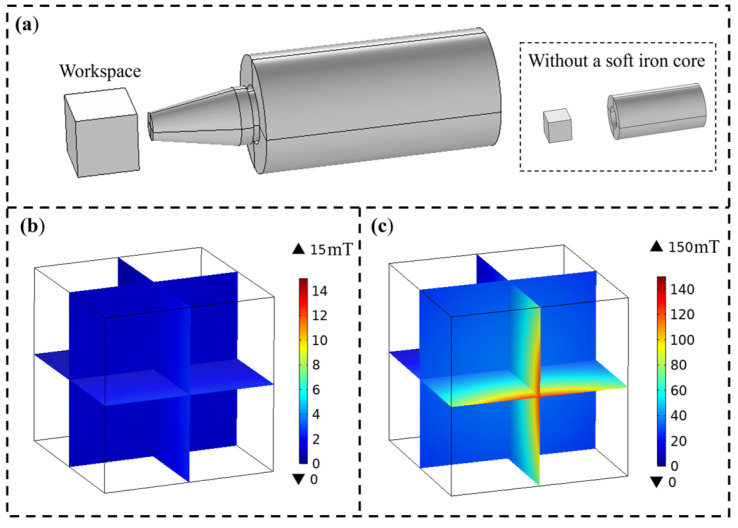
Simulation of a single electromagnetic coil: (**a**) model of a single electromagnetic coil; (**b**) magnetic field distribution in the workspace without a core; (**c**) magnetic field distribution in the workspace with a core.

To study the effect of the iron cores on enhancing the magnetic field of electromagnetic coils and to verify its feasibility, this study employs COMSOL software for magnetic field simulation. Initially, a model of a single electromagnetic coil has been established, as shown in Figure 2a. The simulation grid size of this model ranges from 1 mm to 20 mm. The shape of the finite elements is free tetrahedral, and the number of elements in the mesh is 2,000,190. The simulation of the magnetic field uses the built-in magnetic field module of the software, which calculates the magnetic field values based on Ampere’s law, and the magnetic field in the workspace can be obtained by setting the appropriate parameters. The amount of memory is 29.5 g, and the computing time is 440 s. In the model, a cube with the length of 80 mm represents the workspace, and the electromagnetic coil is applied with a 16 A excitation current. Without a soft iron core, the magnetic field strength produced by the electromagnetic coil is shown in Figure 2b, while with a soft iron core, the magnetic field strength is shown in Figure 2c. It can be found that the magnetic field produced by the electromagnetic coil with a core is significantly stronger than that without a core, thereby confirming that the core can effectively enhance the excitation magnetic field of the electromagnetic coil.

**Figure 3:**
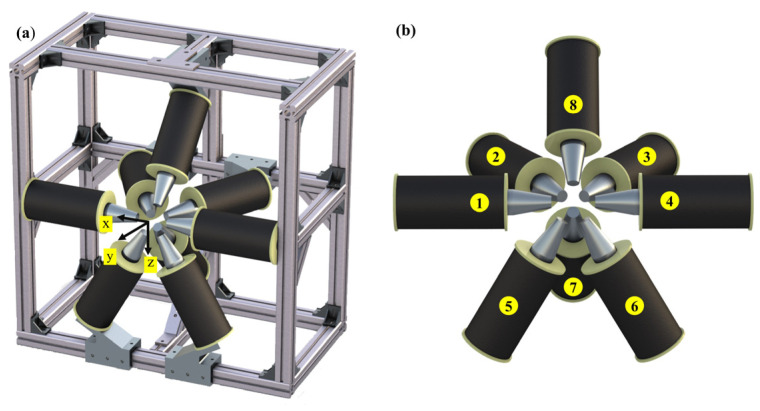
Overall configuration of magnetic field-assisted machining device: (**a**) overall structural framework; (**b**) distribution of electromagnetic coils.

To consider the most basic scenario, controlling the magnetic induction intensity at a specific location in the workspace involves three independent variables, theoretically requiring at least three electromagnetic coils. To control the magnetic field gradient at the target location, five independent variables are needed. Therefore, this study uses eight electromagnetic coils to construct the magnetic field generation device. Additionally, other parts of the device, such as the frame and support blocks, are made from non-magnetic aluminum profiles. As shown in Figure 3, the device is symmetrically distributed in one direction. Electromagnetic coils 1, 2, 3, and 4 are fixed at 60-degree intervals in the xy-plane, electromagnetic coils 1, 4, 5, and 6 are fixed at 60-degree intervals in the xz-plane, and electromagnetic coils 7 and 8 are installed in the yz-plane, each at a 45-degree angle to the xy-plane. The centers of all electromagnetic coils point towards the center of the workspace, which is a spherical region with a diameter of 80 mm centered at this point.

The ease of installing the workpiece during the experiment is a critical issue. While ensuring the magnetic field strength, the distribution of the electromagnetic coils must leave sufficient space for micro electrical machining. To meet this requirement, a special structure is used in this study; that is, the designed magnetic field generation device is interventional, with the magnetic field source occupying less than half of the spherical workspace, preserving external device intervention space. This design facilitates the installation and removal of the setup related to micro electrical machining, allowing the setup to be placed at the center of the spherical region.

#### 2.2.2. Drive System

**Figure 4:**
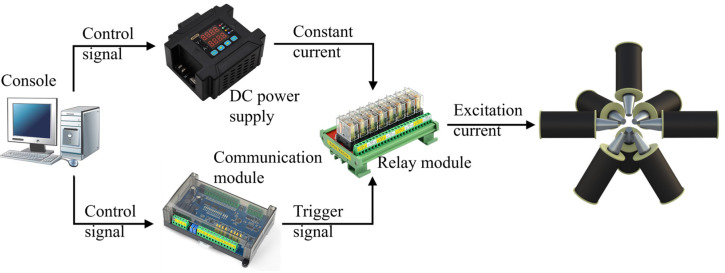
The drive system.

The drive system is shown in Figure 4. Eight constant current power supplies are used to supply power to eight electromagnetic coils, and current control signals are sent to the power modules of each electromagnetic coil through custom communication protocol packets, outputting the calculated excitation current. Relay modules are added to the driving process to achieve current reversal. The Omron BMZ-K1 (Biaokong, Shenzhen, China) series 16-channel relay module is selected, which has an electric load capacity of up to 16 A.

To facilitate control of the relay module by the upper computer PC, the BSM-0808RB communication module is selected. This module utilizes the ModbusRTU communication protocol and supports eight digital or analog inputs and outputs. With a baud rate of 115,200 bps, the communication speed can reach up to 3 ms, meeting the requirements for generating dynamic magnetic fields.

### 2.3. Sampling Device

For the calibration of the model, a large amount of spatial magnetic field data generated by the magnetic field generation device is required. Currently, the magnetic sensors available on the market are basically in the form of probes, and each measurement can only obtain the magnetic field value of a certain direction at a certain point. The measurement process cannot guarantee the accuracy of the measured position and direction, resulting in significant errors and low efficiency. Limited by the above problems, Prof. Song Shuang’s team [18] designed a real-time cubic magnetic sensor array, distributing 64 triaxial magnetic sensors across four planes, with each plane arranging magnetic sensors in a 4 × 4 array. This study has designed a smaller PCB circuit board that encapsulates multiple magnetic sensors and samples the magnetic induction intensity of the workspace layer by layer.

**Figure 5:**
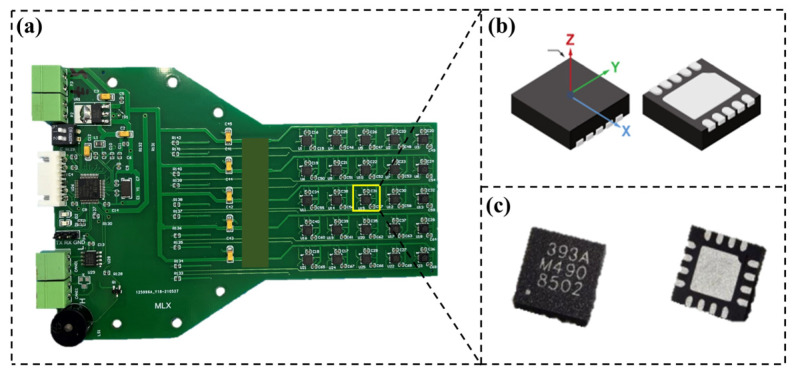
Magnetic field sampling device: (**a**) magnetic field sampling circuit board; (**b**) MLX90393 magnetic sensors; (**c**) ALS31300 magnetic sensors.

This study has selected two different types of magnetic sensors, the MLX90393 (MELEXIS, Ieper, Belgium) and the ALS31300 (Allegro, Manchester, NH, USA) Hall sensors, with their basic information presented in Table 1. The ALS31300 magnetic sensor has a fixed gain and measures within a range of ±100 mT, with a sensitivity of 20 LSB/mT. In contrast, the MLX90393 magnetic sensor offers a dynamic range and programmable gain, with a selectable range of 5~50 mT and a sensitivity range of 311~6211 LSB/mT. When sampling low magnetic field values, the MLX90393 magnetic sensor can be chosen to improve data accuracy. For higher magnetic field values, the ALS31300 magnetic sensor can be used to increase the data sampling range. To improve data sampling efficiency, 25 magnetic sensors are surface-mounted on a PCB, spaced 10 mm apart, as shown in Figure 5. This allows for the sampling of three-dimensional magnetic field values from 25 points within a 40 mm × 40 mm space each time.

The two types of magnetic sensors selected both support the I²C bus communication protocol. The most prominent advantages of the I^2^C bus are its simplicity and effectiveness, allowing data to be transmitted at a maximum rate of 10 Kbps. Each device on the bus has a unique address, enabling communication between the master and slave devices through address selection. Communication between the PCB circuit board and the host computer is conducted using CAN bus, which has a maximum communication rate of up to 1 Mbps and is more suitable for long-distance transmission of large amounts of data.

**Figure 6:**
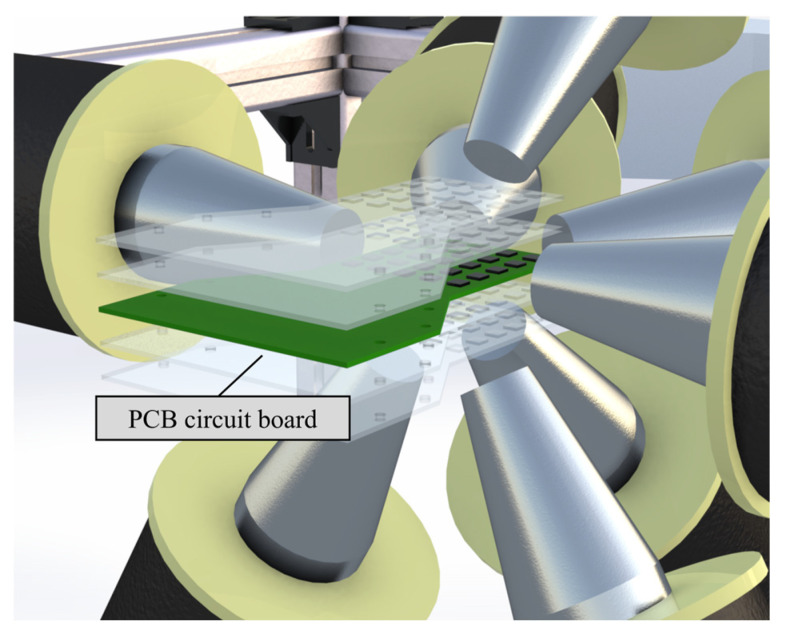
Schematic diagram of the magnetic field sampling scheme.

After applying a set of excitation currents, the PCB circuit board is fixed on an adjustable-height clamp, with each layer of the clamp installed at a height difference of 5 mm. Therefore, with each set of current applied, magnetic field values of 125 spatial points can be sampled in a 5 × 5 × 5 arrangement. The placement of the PCB circuit board during the sampling process is shown in Figure 6.

## 3. Establishment and Calibration of the Mathematical Model

### 3.1. Spherical Harmonic Function Expansion Model

Spherical harmonics are a set of analytical functions defined in spherical coordinates, commonly used to analyze spherical symmetry problems in three-dimensional space. When describing magnetic fields, the spherical harmonics expansion model represents the magnetic field at every point in the spherical coordinate system through these functions. For magnetic fields generated by electromagnetic coils, they can be expanded into a series of sums of spherical harmonics.

#### 3.1.1. Establishment of the Spherical Harmonic Function Expansion Model

Since the magnetic field-generating device is positioned outside the electrolytic machining workspace, we can disregard the current within that space. According to Maxwell’s equations, we have:

(1) $$\left\{\begin{array}{l} \nabla \cdot B=0\\ \nabla \times B=0 \end{array}\right.$$

where ***B*** is the magnetic induction intensity.

Directly using the method of magnetic field numerical interpolation for magnetic field modeling cannot simultaneously satisfy the above two constraints, nor can it handle the significant errors caused by noise during the magnetic field sampling process. Therefore, this study adopts a modeling method based on spherical harmonic function expansion. Since the magnetic field used has no divergence or curl, the magnetic field can be simplified as the derivative of a scalar potential within the magnetic workspace [19]:

(2) B=−∇Φ

where scalar potential Φ is defined by Laplace’s equation:

(3) $$\nabla^{2}\Phi =0.$$

**Figure 7:**
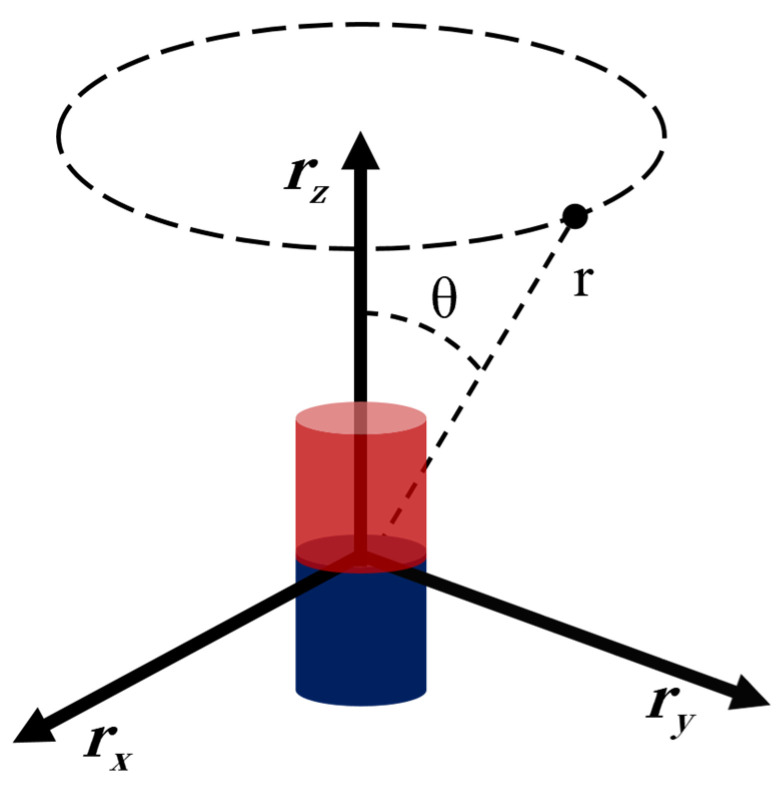
A cylindrically symmetric system in spherical coordinates [19].

The boundary conditions have been described in ref. [19]. In spherical coordinates, the solution to Laplace’s equation for a cylindrically symmetric system, as depicted in Figure 7, is given by the spherical multipole expansion [19]:

(4) $$\Phi \left(r,\cos\left(\theta \right)\right)=\sum_{m=0}^{M}(A_{m}r^{m}+B_{m}\frac{1}{r^{m+1}})P_{m}\left(\cos\theta \right)$$

where $P_{m}\left(\cos\theta \right)$ are Legendre polynomials of order m evaluated at cosθ, r is the distance from the source to the point of interest, and $A_{m}$ and $B_{m}$ are coefficients defined by the boundary conditions of the problem and define the geometric shape of the field attributed to the source.

For systems where the workspace is inside a shell carrying current, like Helmholtz and Maxwell coil pairs, all the $B_{m}$ coefficients must be zero. This is because the field cannot diverge at the center. For systems where the working area is outside a volume enclosing the currents, like solenoid-based systems, all the $A_{m}$ coefficients are zero [19]. This is because the field cannot diverge at infinity. The system adopted in this study is the solenoid-based system, so all coefficients $A_{m}$ are set to zero, and Equation (4) can be simplified as [19]

(5) $$\Phi \left(r,\cos\left(\theta \right)\right)=\sum_{m=0}^{M}B_{m}\frac{1}{r^{m+1}}P_{m}\left(\cos\theta \right).$$

In the magnetic field system, if $r\;=\;p\;-\;p_{s}$, where p is the point in the workspace and $p_{s}$ is the position of the source, then [19]

(6) $$\gamma =\cos\left(\theta \right)=\hat{z}\cdot \hat{r}$$

where $\hat{z}$ is the normalized zenith direction of the spherical potential and $\hat{r}$ is the normalized displacement vector; that is, $\hat{z}=z/\left\|z\right\|$, $\hat{r}=r/\left\|r\right\|$. Therefore, the magnetic induction intensity generated by each magnetic field source can be expressed as [19]:

(7) $$\left\{\begin{array}{l} B\left(r\right)=\sum_{m=1}^{M}\left(C_{2,m}\gamma P_{m}^{\prime }-C_{1,m}P_{m}\right)\hat{r}-C_{2,m}P_{m}^{\prime }\hat{z}\\ C_{1,m}=-\frac{\left(m+1\right)B_{m}}{{\left\|r\right\|}^{m+2}}\\ C_{2,m}=\frac{B_{m}}{{\left\|r\right\|}^{m+2}} \end{array}\right.$$

where $P_{m}$ and ${P_{m}}^{\prime }$ are the Legendre polynomial and the first derivative at γ.

Since Laplace’s equation is linear, the magnetic field is directly related to the excitation current, which also satisfies the superposition principle. The magnetic field can be expressed by the sum of the current weighted contributions of each magnetic field generating source. Assuming a total of S sources, the magnetic field at a certain target position can be expressed as [19]

(8) $$B\left(r\right)=\sum_{s=1}^{S}I_{s}B_{s}\left(r\right)$$

where $I_{s}$ is the excitation current of the electromagnetic coil.

When the excitation current is applied to one or more electromagnetic coils, the coils become magnetized and act as new sources of the magnetic field. Therefore, Equation (8) should be rewritten as [19]

(9) $$B\left(r\right)=\sum_{s=1}^{S}I_{s}\left(B_{s}\left(r_{s}\right)+\sum_{k=1}^{K}B_{s,k}\left(r_{s,k}\right)\right)$$

where $B_{s,k}\left(r_{s,k}\right)$ is the magnetic field generated by the core after the application of a unit excitation current to the electromagnetic coil in the system due to the magnetization of the core.

Considering Equations (5) and (9), it is clear that the essence of establishing a magnetic field mathematical model is to obtain the position of the source, zenith direction, and boundary coefficients of each magnetic field source. If a second-order expansion is used, two boundary coefficients, $B_{1}$ and $B_{2}$, should be included.

#### 3.1.2. Calibration of the Spherical Harmonic Function Expansion Model

Considering the subsequent optimization issues, a second-order field model is used for modeling. The device has eight electromagnetic coils that serve as magnetic field sources. After the excitation current is applied, the eight iron cores are magnetized and become new magnetic field sources. Thus, there are a total of 72 magnetic sources, each including two boundary coefficients, three position parameters, and three direction parameters. In summary, the system designed in this study includes 144 boundary coefficients and 432 spatial parameters (position and direction), totaling 576 parameters to be solved. This is a high-dimensional optimization problem that can be addressed using nonlinear least squares fitting, commonly employing the Newton–Raphson algorithm and the Levenberg–Marquardt (LM) algorithm [20]. In this study, the LM algorithm is used to solve the problem, implemented in C++ language, with the optimization objective function being

(10) $$f_{\mathrm{opt}}=\sum_{m=1}^{M}{\left(B_{\mathrm{measure}}\;-\;B_{\mathrm{model}}\right)}^{2}$$

where $B_{\mathrm{measure}}$ is the measured magnetic flux intensity matrix and $B_{\mathrm{model}}$ is the calculated theoretical magnetic flux intensity matrix.

**Figure 8:**
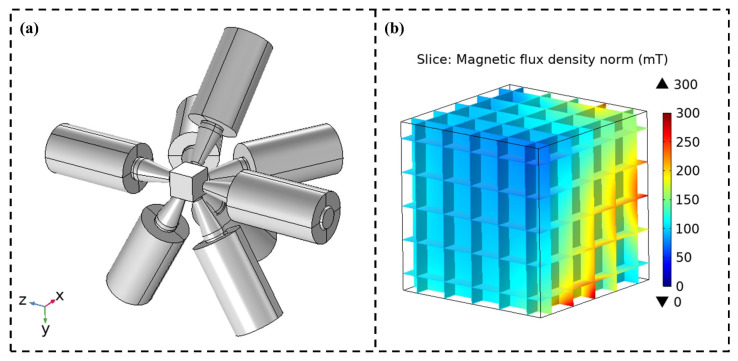
Magnetic field data obtained by simulation: (**a**) simulation model; (**b**) simulation results of single excitation current.

Firstly, the simulation data are used as the $B_{\mathrm{model}}$ for the initial model optimization to obtain the initial model parameters. Similarly, the simulation data are acquired through simulation software, and the established simulation model is shown in Figure 8, with the excitation current group set in sequence, resulting in 3500 sets of magnetic field data. Then, the model is re-optimized using the excitation magnetic field data sampled by the sensors as $B_{\mathrm{measure}}$. Through the final optimization calculation, the obtained magnetic field model has a variance of 0.9977, a root mean square error (RMSE) of 0.71 mT, an error percentage of 2.986%, and average errors in three directions of 0.006347 mT, 0.007565 mT, and 0.01195 mT, respectively. Details of the 144 boundary coefficients and 432 spatial parameters obtained after model calibration can be found in Appendix A.

### 3.2. Magnetic Multipole Superposition Model

The magnetic dipole is a simplified model suitable for describing the magnetic field distribution far from the magnetic source, and it cannot accurately describe the real magnetic field distribution for coils with complex shapes and geometries. However, magnetic quadrupoles and octupoles can more accurately describe the electromagnetic distribution.

#### 3.2.1. Establishment of the Magnetic Multipole Superposition Model

In a certain workspace where no current is passing through, the internal magnetic field can be described using a magnetic scalar potential, and it can be represented as the sum of multipoles [21]. The magnetic field generated by each electromagnetic coil at a unit excitation current is represented as [21]

(11) $$\left\{\begin{array}{l} \begin{array}{l} B\left(r\right)=B_{1}+B_{2}+B_{3}\\ B_{1}=\frac{3\mu_{1}\left({\hat{\mu }}_{1}\cdot \hat{r}\right)}{r^{3}}\hat{r}-\frac{\mu_{1}}{r^{3}}{\hat{\mu }}_{1} \end{array}\\ B_{2}=\frac{3\mu_{2}}{4r^{4}}\left(5{\left({\hat{\mu }}_{2}\cdot \hat{r}\right)}^{2}-1\right)\hat{r}-\frac{3\mu_{2}}{2r^{4}}\left({\hat{\mu }}_{2}\cdot \hat{r}\right){\hat{\mu }}_{2}\\ B_{3}=5\left(\frac{\mu_{3}}{2r^{5}}\right)\left({\hat{\mu }}_{3}\cdot \hat{r}\right)\left[7{\left({\hat{\mu }}_{3}\cdot \hat{r}\right)}^{2}-3\right]\hat{r}-3\left(\frac{\mu_{3}}{2r^{5}}\right)\left[5{\left({\hat{\mu }}_{3}\cdot \hat{r}\right)}^{2}-1\right]{\hat{\mu }}_{3} \end{array}\right.$$

where $B_{1}$, $B_{2}$, and $B_{3}$ are magnetic flux intensity generated by symmetric dipoles, magnetic quadrupoles, and magnetic octupoles, and $\hat{r}$, ${\hat{\mu }}_{1}$, ${\hat{\mu }}_{2}$, and ${\hat{\mu }}_{3}$ are the unit vectors describing the position and the symmetric dipole, magnetic quadrupole, and magnetic octupole moments, respectively.

Similarly, referring to Equation (8), the establishment of a magnetic field mathematical model using the principle of superposition of magnetic multipoles also satisfies a weighted form. Moreover, under this principle, the method of calculating the magnetic field is based on a single electromagnetic coil. Whether it is the magnetic field excited by the solenoid due to the applied excitation current or the magnetic field produced by the magnetization of the soft iron core, both are considered to be generated by the same magnetic source. Therefore, it is only necessary to find the parameters describing the magnetic dipole, quadrupole, and octupole of the electromagnetic coil.

From the analysis of Equation (11), it is known that describing a magnetic pole requires a position vector and a direction vector, with six independent parameters. Since the position vectors for the three types of magnetic poles are the same, describing an electromagnetic coil requires 12 variables, and eight electromagnetic coils have a total of 96 parameters to be solved. Compared to the spherical harmonic expansion, the model based on superposition of magnetic multipoles has far fewer parameters to solve, simplifying the optimization problem.

#### 3.2.2. Calibration of the Magnetic Multipole Superposition Model

This study uses the fmincon function of MATLAB software (R2020a). This function is a nonlinear programming solver that can be used to find the minimum of an objective function under constraints. In this part of the model, it is necessary to define the objective function and the upper and lower bounds of the parameter values.

(12) $$\left\{\begin{array}{c} f_{\mathrm{opt}}=\left|\left.B\right\|\right.F=\mathrm{sqrt}\left(\mathrm{sum}\left(B.*B\right)\right)\\ \begin{array}{c} B=B_{\mathrm{measure}}\;-\;B_{\mathrm{model}}\\ \mathrm{lb}\leq x\leq \mathrm{ub} \end{array} \end{array}\right.$$

where $\left|\left.B\right\|\right.F$ is the Frobenius norm of matrix B, which is the square root of the sum of the squares of all the elements of the matrix. x is the parameter vector containing the position and direction vectors of each magnetic dipole, which are the optimization parameters. lb and ub are the upper and lower limits.

**Figure 9:**
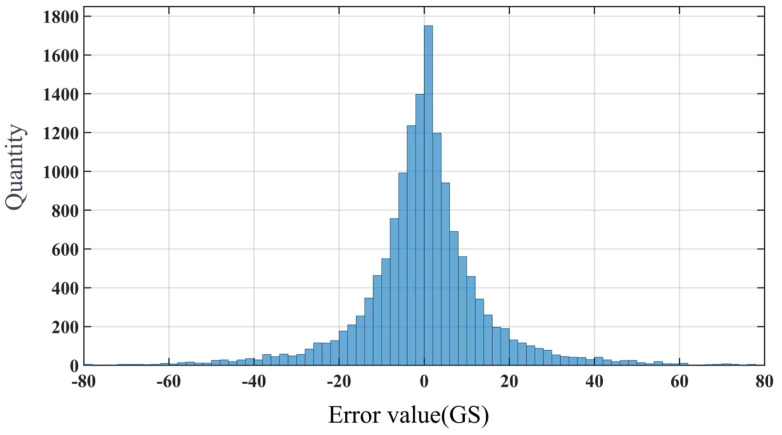
Distribution of model error values after calibration.

Through the optimization, the final magnetic field model has a variance of 0.9935, a root mean square error (RMSE) of 1.67 mT, and an error percentage of 6.981%. The average errors in three directions are 0.01485 mT, 0.01151 mT, and 0.01273 mT, respectively. The distribution of error values, as shown in Figure 9, indicates that the error values are concentrated around zero. The position vectors of each magnetic pole are shown in Table 2, and the direction vectors are illustrated in Table 3.

## 4. Performance Evaluation and Inverse Solution Algorithm

### 4.1. Performance Evaluation

By substituting the parameters collected in practice, we calibrated the theoretical model to make it more in line with the actual situation. The results of the model parameters established using two modeling methods are shown in Table 4. It can be observed that the magnetic field model based on spherical harmonic expansion can more accurately reflect the magnetic field distribution in the workspace. This outcome suggests that if high precision is required for the magnetic field model in practical applications, the spherical harmonic expansion is a better choice.

Although this approach has a higher computational complexity, the precision of the calculations can be improved by increasing the number of terms in the spherical harmonic expansion. In contrast, the accuracy of the magnetic multipole model is limited by its inherent characteristics. Therefore, subsequent algorithms for calculating the reverse driving current and control algorithms will be based on the model from the spherical harmonic expansion.

It is necessary to define performance indicators for evaluation, such as magnetic field strength, magnetic field gradient, and uniformity. During the experimental process, errors caused by the installation of fixtures and workpieces can lead to discrepancies between the actual processing position and the target position set by the magnetic field. To reduce the experimental errors caused by the aforementioned situations, it is essential to ensure that there are no significant changes in the magnetic field within the workspace. Additionally, it is crucial to ensure that the magnetic flux intensity at the working position does not vary significantly. For the field, the uniformity index is commonly considered for evaluating uniformity, which is defined as [5]

(13) $$\gamma \left(\varphi \right)=1-\frac{\sum_{p}^{N}\left|\varphi \left(p\right)-\langle \varphi \rangle \right|}{2N\langle \varphi \rangle }$$

where *N* is the number of samples, $\varphi \left(p\right)$ is the magnetic field strength at position p, and $\langle \varphi \rangle$ is the average magnetic field of the sample, expressed as

(14) $$\langle \varphi \rangle =\frac{1}{N}\sum_{p}^{N}\varphi \left(p\right)$$

The calculation of the uniformity index involves the magnetic field values of the workspace sampled in practice. These values are then substituted into Equation (13), resulting in a uniformity index of 92.17%. This high uniformity index indicates that the variation in magnetic flux intensity is relatively smooth at every position within the workspace. A highly uniform magnetic field can effectively reduce the magnetic field fluctuations caused by errors in fixture and workpiece installation, thereby improving the stability and repeatability of the experiments.

### 4.2. Inverse Solution Algorithm

As indicated by Equation (8), the magnetic field at a certain point in the workspace is the sum of contributions from electromagnetic coils. The magnetic flux intensity in the workspace can be represented as

(15) $$B\left(P\right)=\sum_{n=1}^{N}B_{n}\left(P\right)=\sum_{n=1}^{N}{\tilde{B}}_{n}\left(P\right)i_{n}$$

where $i_{n}$ is the excitation current of the electromagnetic coil, P is a target position vector in the workspace, $B_{n}\left(P\right)$ is the magnetic induction intensity generated by the electromagnetic coil at position P, and ${\tilde{B}}_{n}\left(P\right)$ is the magnetic induction intensity generated by the electromagnetic coil at position P under unit current excitation. Equation (15) can be written in matrix form as

(16) $$B\left(P\right)=\left[\begin{array}{ccc} {\tilde{B}}_{1}\left(P\right) & \cdots & {\tilde{B}}_{n}\left(P\right) \end{array}\right]\left[\begin{array}{c} i_{1}\\ \vdots \\ i_{n} \end{array}\right]=\beta \left(P\right)I$$

where $\beta \left(P\right)$ is defined as the unit current drive matrix, which is a 3 × *n* matrix. For convenience, use $A\left(P\right)$ instead of $\beta \left(P\right)$. Therefore, the inverse solution equation of the current set can be expressed as

(17) $$I=A{\left(P\right)}^{†}\cdot B{\left(P\right)}_{\mathrm{target}}$$

where $A{\left(P\right)}^{†}$ is the pseudo-inverse of $A\left(P\right)$ and $B{\left(P\right)}_{\mathrm{target}}$ is the target magnetic induction matrix.

As indicated by Equation (17), the solution for the current group is not unique, making the reverse solution of the driving current group a nonlinear optimization problem. This study uses the open-source nonlinear optimization library NLopt (Nonlinear Optimization), which supports multiple languages and features multi-parameter and multi-constraint optimization, as well as support for equality, local and global optimization, inequality, and bounded constraints.

In this system, there are eight electromagnetic coils, and the maximum current value for each coil is determined by the constant current power supply, thus setting 16 A as the current upper limit. All magnetic field matrices are represented as 3×1 matrices. Based on the above conditions, the following expression can be established:

(18) $$\left\{\begin{array}{c} {\min\;f(i)=i}_{1}^{2}{+i}_{2}^{2}{+i}_{3}^{2}{+i}_{4}^{2}{+i}_{5}^{2}{+i}_{6}^{2}{+i}_{7}^{2}{+i}_{8}^{2}\\ \left[\begin{array}{c} \beta_{x}\left(P\right)\\ \beta_{y}\left(P\right)\\ \beta_{z}\left(P\right) \end{array}\right]I=B_{\mathrm{target}}\\ \;-\;16\leq i_{n}\leq 16\left(I_{\max}\right) \end{array}\right.$$

where f(i) is the objective function, and $\beta_{x}\left(P\right)$ is the component of $\beta \left(P\right)$ in the x direction, which is a 1 × 8 matrix, and the same goes for $\beta_{y}\left(P\right)$ and $\beta_{z}\left(P\right)$.

To verify the feasibility and system performance of the algorithm, 20 sets of excitation currents are applied sequentially, and the magnetic flux intensity in the workspace is obtained using a magnetic field sampling device. The sampled data are compared with the calculated data from the mathematical model of the magnetic field, resulting in average errors in three directions of 0.00782 mT, 0.00833 mT, and 0.01109 mT, respectively.

To validate the magnetic field control performance of the device, a magnetic field driving experiment has been conducted on a small cylindrical magnet. The cylindrical magnet had a diameter of 1 mm and a thickness of 5 mm, with the magnetization direction along the thickness. Initially, the cylindrical magnet is placed in the workspace using a soft rope traction method. Subsequently, the target position excitation magnetic field is set, and the driving currents for the eight electromagnetic coils are obtained through the reverse calculation algorithm.

**Figure 10:**
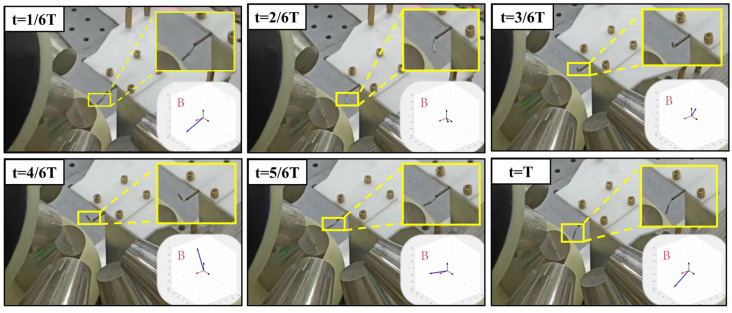
Magnetic field manipulation of a small magnet.

To ensure the real-time and accuracy of dynamic magnetic field control, the host computer is set to send a driving current command to the control hardware every 25 ms. In this paper, the response frequency limit of the presented electromagnet has not been tested. In the experiment, the target dynamic magnetic field is set as a conical pendulum magnetic field, and the results are shown in Figure 10. By comparing the orientation of the cylindrical magnet at each instant with the set target magnetic field direction, we can observe that the two orientations are generally consistent, strongly verifying the performance and accuracy of the system.

## 5. Conclusions

In this study, a magnetic field-assisted platform has been developed to investigate the effects of a magnetic field on micro electrical machining. The device can generate magnetic fields in any direction within the workspace with intensities ranging from 0 to 0.2 T.

To accurately describe the magnetic field in the workspace, this study investigates two models: the spherical harmonic expansion and the multipoles superposition. Additionally, a magnetic field sampling system has been designed to gather magnetic field data, and based on which the magnetic field model has been calibrated and optimized. The final magnetic field model arrives at an error percentage of 2.986%, a variance of 0.9977, and a root mean square error (RMSE) of 0.71 mT, realizing precise characterization of the workspace’s magnetic field. Based on the established magnetic field model, a reverse calculation algorithm for the driving current is completed, achieving control over the magnetic field in the workspace. Finally, this study presents simple driving experiments to indicate the controllability of the magnetic field generated by the proposed system.

## Figures and Tables

**Table 1:** Parameters of magnetic sensors.

Type	Measurement Range (mT)	Gain Control Function	Sensitivity (LSB/mT)	Measurement Dimension	Output Data (bit)
MLX90393	5–50	Yes	311–6211	Three axes	12
ALS31300	100	No	20	Three axes	16

**Table 2:** The magnetic pole position parameters after optimization.

Number	1	2	3	4	5	6	7	8
$r_{x}$ (m)	0.0033	0.0685	0.0725	0.0256	−0.0013	−0.0044	0.0499	0.0830
$r_{y}$ (m)	0.0074	−0.018	−0.0019	0.0234	0.0712	0.0695	0.0661	−0.0285
$r_{z}$ (m)	0.0974	0.0502	−0.0437	−0.0665	0.0443	−0.0451	0.0012	0.0027

**Table 3:** The magnetic pole direction parameters after optimization.

Number	1	2	3	4	5	6	7	8
$\mu_{1x}$	5.837	5.800	3.972	−11.831	−11.551	−13.945	−2.017	−7.145
$\mu_{1y}$	−10.782	−4.496	−17.726	−8.996	−0.345	−3.796	11.462	−6.575
$\mu_{1z}$	−6.196	7.456	1.723	3.368	0.256	−1.329	1.188	0.037
$\mu_{2x}$	−4.353	2.008	−2.043	−0.761	0.389	0.726	−0.903	−1.201
$\mu_{2y}$	0.716	−0.041	1.838	−0.512	−0.822	−0.641	−1.757	0.290
$\mu_{2z}$	−2.954	1.278	0.412	−0.454	−0.461	0.725	−0.099	−0.051
$\mu_{3x}$	−0.163	−0.025	−0.002	0.005	0.003	−0.007	0.011	0.050
$\mu_{3y}$	0.053	−0.042	0.042	−0.001	0.012	−0.001	0.066	−0.037
$\mu_{3z}$	0.035	0.015	−0.027	−0.002	−0.006	−0.013	0.001	−0.005

**Table 4:** Results of the mathematical model of magnetic field.

	Spherical Harmonic Function Expansion	Multipole Superposition
RMSE (mT)	0.71	1.66
error percentage (%)	2.986	6.981
$R^{2}$	0.9977	0.9935

## Data Availability

The original contributions presented in the study are included in the article, further inquiries can be directed to the corresponding author.

## Appendix A. Model Optimization Results

Based on the expansion of spherical harmonic functions, the optimization results for the model parameters are as follows. After the optimization of the system mathematical model, 144 boundary coefficients and 432 spatial parameters for 72 magnetic sources were obtained. In this context, B1 and B2 represent boundary parameters, $p_{s}\left(x\right)$ represents the component of the position parameter in the x-direction, and $z\left(x\right)$ represents the component of the zenith direction in the x-direction. Other parameters are analogous.

**Table A1:** The optimization results based on the spherical harmonic function expansion model.

B1	B2	$p_{s}\left(x\right)$	$p_{s}\left(y\right)$	$p_{s}\left(z\right)$	$z\left(x\right)$	$z\left(y\right)$	$z\left(z\right)$
1.852 × 10^−6^	4.786 × 10^−8^	0.0698	0.0477	−0.0137	0.6597	0.7502	−0.0456
−3.356 × 10^−7^	−2.175 × 10^−8^	0.0108	0.0396	−0.0813	0.3181	0.9303	−0.1826
−1.463 × 10^−5^	−2.267 × 10^−7^	0.0117	0.0188	0.1031	0.2565	0.8825	0.3941
4.173 × 10^−6^	1.075 × 10^−7^	0.0032	0.0048	0.0796	0.1477	0.2348	0.9608
2.114 × 10^−8^	1.603 × 10^−10^	0.0347	−0.0394	0.0053	0.6810	−0.6341	0.3662
1.254 × 10^−7^	−4.952 × 10^−14^	0.0525	0.0042	0.0334	0.8276	0.4058	−0.3878
−3.020 × 10^−5^	4.242 × 10^−6^	0.0482	0.0301	0.1828	−0.1998	−0.8720	0.4468
−2.038 × 10^−10^	−2.123 × 10^−7^	−0.0033	0.0444	0.0470	−0.1278	0.1424	0.9815
−9.315 × 10^−7^	1.861 × 10^−8^	0.0303	0.0750	−0.0452	−0.7342	0.3069	−0.6056
3.289 × 10^−6^	−2.146 × 10^−7^	0.0488	0.0725	0.0169	−0.0476	−0.6596	0.7501
−2.277 × 10^−5^	1.545 × 10^−6^	0.0718	0.0045	−0.1313	0.2069	−0.5240	0.8262
−4.089 × 10^−6^	9.826 × 10^−7^	0.0482	0.0265	0.0947	0.7458	0.6565	0.1132
−4.024 × 10^−6^	−7.492 × 10^−7^	0.0913	0.0075	0.0504	0.5464	−0.0004	−0.8375
−2.234 × 10^−7^	−1.460 × 10^−13^	0.0547	−0.0352	0.0067	−0.7220	0.2404	0.6488
−2.204 × 10^−7^	−6.420 × 10^−7^	0.0443	−0.0147	0.0502	0.4075	0.3974	0.8222
9.224 × 10^−6^	2.287 × 10^−10^	0.0321	0.0199	0.0811	0.5022	0.8415	−0.1992
6.717 × 10^−6^	1.697 × 10^−7^	0.0478	0.0607	0.0267	0.6202	0.7754	−0.1186
4.741 × 10^−5^	−7.232 × 10^−6^	0.1116	0.0198	−0.1557	0.3856	−0.5686	0.7266
−1.727 × 10^−5^	−8.702 × 10^−7^	0.0738	−0.0122	−0.0524	0.4298	0.5317	−0.7297
−4.331 × 10^−6^	6.224 × 10^−7^	0.0467	0.0237	−0.0946	−0.2567	−0.7893	−0.5578
4.592 × 10^−6^	2.293 × 10^−7^	0.0384	0.0678	−0.0160	0.9925	0.0813	−0.0914
−1.777 × 10^−6^	−1.627 × 10^−8^	0.0351	0.0529	0.0028	0.4392	0.7355	−0.5160
−1.254 × 10^−6^	−3.332 × 10^−7^	0.0717	−0.0272	−0.0259	0.3902	0.3405	−0.8554
8.302 × 10^−6^	7.296 × 10^−7^	0.0834	−0.0276	−0.0207	0.7731	0.2892	−0.5645
−2.976 × 10^−6^	1.502 × 10^−6^	0.0736	0.0104	−0.0815	0.7901	−0.5037	0.3493
2.347 × 10^−6^	1.307 × 10^−7^	0.0378	0.0632	−0.0164	0.1541	0.9362	0.3160
−1.454 × 10^−7^	−1.083 × 10^−8^	0.0306	−0.0142	0.0818	0.8138	−0.3038	0.4954
3.052 × 10^−5^	1.829 × 10^−6^	−0.0053	0.0265	−0.1096	−0.1837	0.5165	−0.8363
−2.241 × 10^−6^	−4.479 × 10^−8^	−0.0414	0.0448	−0.0571	0.1105	0.3891	−0.9145
1.376 × 10^−5^	2.284 × 10^−8^	−0.1551	0.0433	0.0025	−0.3551	0.6283	0.6923
7.440 × 10^−8^	−3.685 × 10^−7^	0.0486	−0.0513	−0.0245	0.1860	−0.3111	−0.9320
−2.283 × 10^−7^	1.303 × 10^−8^	0.0577	0.0095	−0.0450	−0.3503	0.6881	0.6355
−1.054 × 10^−4^	−9.663 × 10^−6^	−0.1504	−0.0692	−0.1045	−0.6370	0.1212	−0.7613
1.512 × 10^−4^	−2.765 × 10^−5^	0.2174	0.0112	−0.0827	−0.6203	−0.7406	−0.2582
−3.677 × 10^−6^	9.614 × 10^−8^	0.0651	0.0575	−0.0472	0.8263	−0.2458	0.5068
5.837 × 10^−6^	4.996 × 10^−7^	0.1177	0.0170	0.0406	0.4758	0.5828	0.6588
1.625 × 10^−5^	3.578 × 10^−7^	0.0333	0.0754	−0.0272	0.3182	0.7283	−0.6070
−7.833 × 10^−6^	2.160 × 10^−7^	0.0331	0.0776	−0.0544	0.3613	0.4606	−0.8108
5.153 × 10^−6^	6.177 × 10^−7^	−0.0508	0.0196	0.0854	−0.1789	−0.3363	−0.9246
1.361 × 10^−5^	−7.680 × 10^−7^	0.0749	0.0322	0.0622	0.5451	−0.7035	−0.4560
−3.531 × 10^−6^	−9.370 × 10^−8^	0.0658	0.0448	−0.0305	0.2611	0.9635	−0.0598
8.517 × 10^−6^	2.812 × 10^−7^	0.0725	0.0184	0.0514	−0.0971	0.7650	0.6367
9.883 × 10^−6^	6.359 × 10^−8^	−0.0521	0.0121	0.0701	0.2541	0.1740	0.9514
−1.207 × 10^−5^	−4.261 × 10^−7^	0.0005	0.0857	0.0466	0.8180	0.4105	0.4028
−8.291 × 10^−7^	4.109 × 10^−10^	0.0427	−0.0511	0.0040	−0.4143	−0.2761	−0.8672
−8.420 × 10^−8^	2.511 × 10^−11^	0.0362	0.0118	−0.0459	0.8318	−0.2021	0.5170
−7.135 × 10^−6^	−6.314 × 10^−7^	0.0213	0.0495	−0.1078	0.3477	0.4793	−0.8058
6.473 × 10^−7^	2.034 × 10^−8^	−0.0171	0.0761	0.0128	0.5871	0.7730	−0.2404
4.401 × 10^−6^	7.607 × 10^−8^	0.0502	0.0367	−0.0005	0.4099	−0.2998	−0.8615
−2.122 × 10^−8^	−1.026 × 10^−7^	0.0593	−0.0280	0.0013	0.8891	0.4388	0.1306
4.286 × 10^−6^	−6.836 × 10^−8^	0.0500	0.0362	−0.0004	−0.4230	0.2836	0.8606
−5.609 × 10^−5^	−7.197 × 10^−6^	−0.0644	−0.1493	0.2044	−0.1933	−0.5460	0.8152
−2.833 × 10^−6^	−9.181 × 10^−8^	−0.0044	0.0707	−0.0434	0.8192	0.5451	−0.1786
1.251 × 10^−7^	−5.794 × 10^−7^	0.0010	0.0256	0.0718	−0.1266	−0.8044	−0.5804
3.407 × 10^−5^	8.510 × 10^−7^	0.0501	0.0639	−0.0597	0.6182	0.7481	−0.2411
5.812 × 10^−4^	−6.535 × 10^−6^	0.0160	0.2134	−0.1578	0.9863	0.1606	−0.0389
1.032× 10^−3^	6.605 × 10^−5^	0.1046	0.1774	0.1223	0.1763	−0.3632	−0.9149
5.091 × 10^−5^	1.290 × 10^−6^	0.0642	0.0507	0.0604	0.7504	0.6101	0.2543
7.110 × 10^−5^	3.165 × 10^−6^	0.0427	−0.0449	−0.1256	0.5227	−0.1085	−0.8456
3.336 × 10^−5^	7.948 × 10^−7^	0.0941	0.0150	−0.0084	0.8902	0.4450	−0.0972
1.061 × 10^−4^	5.742 × 10^−6^	0.0925	−0.1154	0.0509	0.8438	−0.4726	0.2542
5.430 × 10^−5^	1.417 × 10^−6^	0.0201	0.1022	0.0113	0.6045	0.7839	0.1420
6.705 × 10^−6^	2.280 × 10^−7^	−0.0815	−0.0779	−0.0031	−0.7106	−0.6982	−0.0871
2.785 × 10^−6^	3.386 × 10^−8^	0.0602	−0.0327	−0.0331	−0.7289	−0.6841	−0.0255
−9.890 × 10^−6^	−8.819 × 10^−7^	0.0451	0.0964	−0.1206	0.5812	0.0786	−0.8100
−1.453 × 10^−5^	−4.015 × 10^−7^	0.0100	0.0066	0.1051	0.2996	0.2979	0.9064
4.225 × 10^−6^	1.039 × 10^−7^	0.0474	−0.0370	0.0295	−0.1598	−0.9525	−0.2591
−3.992 × 10^−6^	−5.271 × 10^−7^	0.0476	−0.0508	−0.0483	−0.2556	−0.5166	−0.8172
3.676 × 10^−6^	−1.225 × 10^−7^	0.0480	−0.0379	0.0294	0.2667	0.9300	0.2528
3.351 × 10^−6^	8.210 × 10^−7^	0.0487	−0.0548	−0.0518	0.5609	0.4584	0.6893
1.415 × 10^−5^	−8.976 × 10^−6^	0.0662	0.0309	0.1660	0.5868	0.3099	0.7481
3.708 × 10^−5^	2.469 × 10^−8^	0.1169	0.0297	0.0940	−0.8309	−0.2987	0.4695
